# Blood pressure measurement practices in children and adolescents within primary care setting

**DOI:** 10.3389/fped.2025.1571419

**Published:** 2025-03-21

**Authors:** Kamilė Čeponytė, Karolis Ažukaitis, Augustina Jankauskienė

**Affiliations:** Faculty of Medicine, Vilnius University, Vilnius, Lithuania

**Keywords:** blood pressure, measurement technique, children, adolescents, primary care setting

## Abstract

**Objective:**

Poor compliance to the technical aspects of blood pressure (BP) measurement procedure may lead to inaccurate estimation of BP and misclassification of patients. However, the latter have not been explored systematically. We aimed to assess real-life BP measurement practices in Lithuanian children and adolescents at the primary care setting, and their compliance with current European Society of Hypertension (ESH) guidelines.

**Methods:**

Two cross-sectional surveys were conducted in Lithuania. The study population included parents, who have children aged 0–17 years, and was further enriched by adolescents aged 14–17 years. Original questionnaires were developed and used to survey the participants.

**Results:**

Study included 1,504 parents and 448 adolescents. Median age of the surveyed parents' children and adolescents was 6 years and 50.2 percent were female. Overall, among all children aged 3 years or older only 55% of respondents reported BP measurements at least once. The rates of BP measurements increased with age and exceeded 80 percent from 14 years. Only 3.3 percent of respondents reported no issues with BP measurement procedure. The most common errors included single measurements of BP (81.4%), lack of feedback (60.2%), incorrect positioning (40.7%), miscuffing (39.2%) and lack of rest period (27.9%).

**Conclusions:**

Our study reveals not only insufficient BP screening rates within Lithuanian primary care setting, but also high rates of technical errors during BP measurement procedure. Collectively, these issues likely contribute to misdiagnosing of arterial hypertension and suboptimal care of children who are at risk of inaccurate and imprecise BP results.

## 1 Introduction

Increasing prevalence of arterial hypertension (AH) is one of the foremost public health problems in children and adolescents (1, 2). Elevated blood pressure (BP) in young age has been shown to track into adulthood and associate with adverse cardiovascular outcomes (3). Children diagnosed with AH have higher risk of premature death caused by cardiovascular disease (4). Thus, to relieve the burden of cardiovascular disease in adults it is important to diagnose, manage and start treatment as early as possible in order to prevent hypertension-mediated organ damage development and to improve cardiovascular outcomes. Screening for hypertension in children and adolescents typically involves office BP measurements, that require minimal cost and time (5–7). European Society of Hypertension (ESH) 2016 guidelines for the management of high BP in children and adolescents layout the principles of BP screening, including measurement at rest, repeated measurements, proper positioning, appropriate cuff size, and the use of validated devices (8). Although prior studies have well described that the adherence to pediatric BP screening and management guidelines in generally poor, the compliance to technical requirements of BP measurement procedure has not been widely explored (9–11).

The aim of this study was to determine the extent of arterial BP measurements in children and adolescents, and the compliance to best practice recommendations for BP measurement procedure in the real-world setting within the primary care settings in Lithuania.

## 2 Materials and methods

### 2.1 Study design and participants

Two cross-sectional surveys were conducted in Lithuania. Parents were surveyed from 2022 October 4th to 2023 January 23rd using Google Forms via social media platforms (the survey was advertised in various popular parental social media groups). In order to enrich the cohort with older children, hard copies of questionnaire were distributed to 14–17-year-old adolescents at various schools and non-formal education centers from October 2023 to March 2024.

### 2.2 Questionnaire

Original questionnaires were constructed according to the 2016 ESH guidelines for the management of high BP in children and adolescents and best practice recommendations (8, 12). Questionnaires for parents and adolescents consisted of 15 single choice questions related to demographic characteristics, comorbidities and life history, children and adolescents BP measurement practices in the primary care setting (Supplementary Material S1 and S2). The clarity of questionnaires was evaluated by conducting a pilot study with 30 participants (15 parents and 15 adolescents) in outpatient clinic. During this process parents and adolescents attending outpatient consultations received paper copies of the survey and were asked in person to provide feedback on any unclear terms or phrases. Accordingly, corrections were made based on their feedback.

### 2.3 Data analysis

The extent of BP measurement in primary care setting and BP measurement practices in children were analysed using MS Excel, and IBM SPSS v.29 tools. The normality of the quantitative data was tested using the Shapiro–Wilk test. Non-normally distributed data variables were presented as median (min-max). Qualitative variables were expressed as frequencies and percentages and presented as *n* (%).

## 3 Results

1,504 parents and 448 adolescents answered the questionnaire. Median age of the surveyed parents' children and adolescents was 6 (0.06–17) years and half were female. Respondents were from more than 26 different districts across all Lithuania. Self-reported diagnosis of AH was indicated in nearly two percent of all children and adolescents (*n* = 1,952). Baseline characteristics of the two groups are presented in Table 1.

**Table 1:** Parents’ and adolescents reported data: baseline characteristics.

Characteristic	*n* = 1,952
Girls, *n* (%)	979 (50.2)
Age, median (min-max)	6 (0.06–17)
Born premature or admitted to neonatal intensive care unit, *n* (%)	260 (13.3)
Comorbidities, *n* (%):	104 (5.3)
Congenital heart disease	71 (68.3)
Chronic kidney disease	7 (6.7)
Diabetes mellitus	9 (8.7)
Other (e.g., asthma, cystic fibrosis, hydronephrosis)	17 (16.3)
Self-reported diagnosis of AH, *n* (%)	38 (1.9)

**Figure 1:**
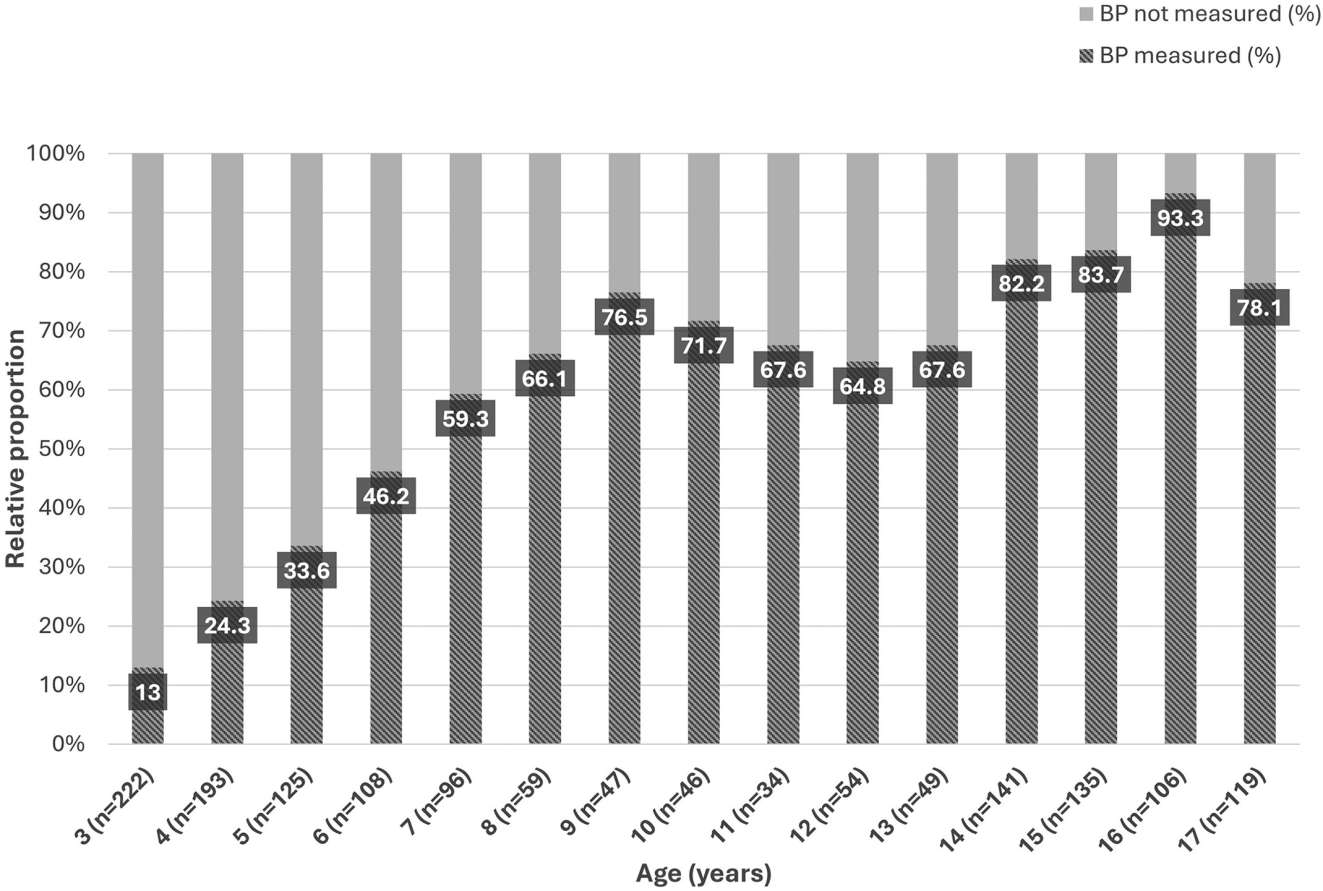
Arterial blood pressure measurements by age.

**Figure 2:**
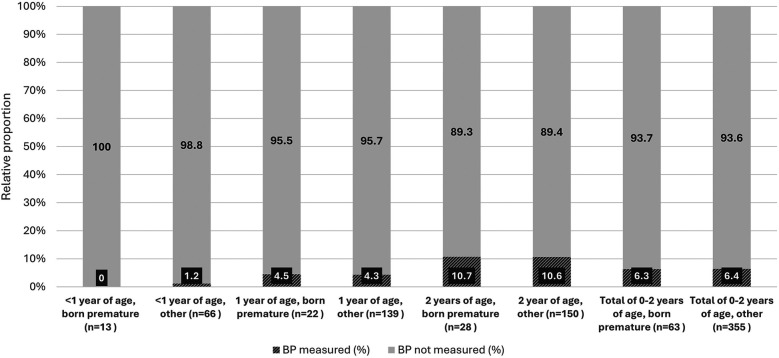
Arterial blood pressure measurements first two years of life.

Overall, less than half of the children and adolescents had their BP measured at least once in the primary care setting. Of those who were eligible for BP screening (from 3 years of age) according to the ESH guidelines (*n* = 1,534), 845 (55%) had their BP measured at least once. The proportion of children and adolescents reported to have had their BP measured increased with age and exceeded 50 percent since school age (Figure 1). Majority of those (656, 77.7%) had their BP measured annually, 104 (12.3%) less than once a year and 85 (10%) more frequently (*n* = 845). Among children younger than 3 years of age (*n* = 418), BP was measured at least once in 27 (6.4%) (Figure 2). Overall, 63 (15%) of these children were born premature and only 4 of them (6.3%) had their BP measured at least once.

Data summarizing BP measurement practices for all children and adolescents who had their BP measured (*n* = 1,952) are presented in Table 2. Only 66 (3.3%) of the respondents reported full compliance to the recommended BP measurement practices according to the ESH guidelines and best practice recommendations. Automated BP measurement devices were used in slightly more than half of children and adolescents who had their BP measured.

**Table 2:** BP measurement practices in the primary care setting.

Practice	Children and adolescents who had their BP measured at least once (*n* = 872)
BP measured at rest, *n* (%)	629 (72.1)
Appropriately sized upper arm cuff used, *n* (%)	531 (60.8)
BP measured only one time during the visit, *n* (%)	710 (81.4)
Incorrect positioning of the child during measurements, *n* (%)	355 (40.7)
BP measured using automated device/auscultatory method, *n* (%)	464 (53.2)/408 (46.8)
Physician feedback on BP results to the parents and/or children, *n* (%)	347 (39.8)

BP was measured at rest for most children and adolescents. Appropriate upper arm cuff size selection was indicated by more than sixty percent of respondents. Incorrect positioning (i.e., not sitting straight, feet not resting on the floor, arm not relaxed, or upper arm cuff not placed at the same level as the heart) during BP measurements was observed by more than forty percent. The most frequent mistakes in BP measurement for children and adolescents were—only one measurement per visit and lack of feedback. More detailed and separated data of parents' and adolescents' can be found in Supplementary Tables S3 and S4.

## 4 Discussion

In the present article we aimed to explore real-life practices of BP measurement in children and adolescents within the primary care setting across different age groups with a focus on compliance to technical requirements as suggested by the best practice recommendations. Our data from the survey of parents and adolescents indicate that less than half of children and adolescents had their BP measured in the primary care at least once with the lowest rates in pre-school children. Moreover, we sought to explore whether BP measurement is performed in compliance to the ESH guidelines and best practice recommendations and found that only few percent of survey participants indicated no errors in the measurement procedure.

Measurement of BP has already been long recognized as an essential part of routine pediatric physical examination, but practice still remains inconsistent. Even though, BP measurement is a relatively quick, cost-effective and non-invasive method for screening of hypertension in the pediatric population (13), the underdiagnosing of AH in children is still widely reported and attributed to challenges in BP measurement, difficulties in recognizing elevated BP, and primary care physicians' unfamiliarity with the guidelines (14–16). The data suggests that pediatric hypertension is largely underdiagnosed, with only approximately of one quarter of children with abnormal BP diagnosed with AH and frequently lacking appropriate follow-ups (17, 18). In addition, a prior analysis of the Lithuanian health registries revealed the prevalence of AH diagnosis in the electronic health system to be 0.29% among children aged 0–17 years, suggesting that AH in children might be underdiagnosed (19).

Moreover, beyond under-recognition of abnormal BP measurements, even a larger issue may be related to the lack of appropriate screening. A 2013 survey of general practitioners highlighted the limited resources for pediatric BP measurement in primary care and noted that routine BP assessments are often deferred until children approach adulthood (20). Although positive trends have been reported over the last two decades in BP measurement availability, nurse involvement, and technology use (21), available data suggest that this issue still remains relevant. Analysis of electronic health records from the Canadian primary care sector revealed that only one-third of the pediatric encounters contained documented BP readings (22). Similarly, suboptimal BP measurement practices have been observed in pediatric assessment unit patients and in general pediatric wards, with only one-third and just over a half of patients with recorded BP, respectively (23). Finally, a study by, Tsoumakas et al. found that 47.2% of children in Greece never had their BP measured, with 55.3% of those exhibiting prehypertension or hypertension lacking prior checks, often due to parental unawareness (24). Our findings indicating that only 55% of children older than 3 years reported BP measurements at least once, are in line with prior studies. Also, our data are in line with prior observations that younger children are less likely to undergo BP measurements than adolescents (25).

Even when BP is measured, the correct BP measurement procedure is critical to ensure reliable readings and to avoid potential misclassification of BP status. Current ESH guidelines for the management of high BP in children and adolescents outline recommendations for proper BP measurement procedure, including among others: setting, positioning, cuff choice and method of measurement. Overall, only 3.3 percent of respondents reported full compliance to the measurement procedure recommended in the guidelines with varying rates regarding different recommendations. This number is strikingly similar to the findings of Rea et al. who evaluated the adherence to clinical practice guidelines and found that only two percent of children with high BP had all BP measurements steps completed correctly (10).

Generally, a 3–5 min rest is recommended before the start of BP measurement and insufficient rest period has been reported to result in 4.2–11.6 mmHg higher systolic BP readings (26). In our study, only slightly more than 70 percent of respondents reported that their BP was measured at rest. Although a randomized controlled trial in the adult population stated that shorter rest periods result in minimal difference (within ± 2 mmHg) in those with normal BP (27), robust evidence from the pediatric population are lacking. However, considering that children generally show higher sympathetic nervous system (SNS) activity that decreases with age (28), insufficient rest period may result in palpable difference, particularly in younger children where even small errors in BP reading may lead to misclassification.

Insufficient resting period is particularly important considering that strikingly over 80 percent of respondents reported that only single measurement of BP was performed during an encounter. A systematic review on sources of error in BP measurements reported that relying on a single BP measurement may result in overestimation of systolic BP by 3.3–10.4 mmHg (26). In the pediatric population, a study by Outdili et al. found that using only the first measurement of BP results in the lowest discrimination for hypertension compared to the mean of first two or second and third measurements (29). Specifically, using only the first measurement resulted in 80 percent specificity for the diagnosis of AH. This can be translated into up to 16 percent of our studied population being at risk of misclassification as hypertensive only due to the lack of repeated BP measurements.

Appropriate cuff size selection is another condition which is known to affect accuracy of BP measurements. ESH guidelines recommend using cuffs with bladder width that is 40% of the arm circumference and length that covers 80%–100% of the circumference. Data from a randomized controlled trial in adult population suggested that overcuffing or undercuffing resulted in striking under- and overestimation of systolic BP readings, respectively (30). Study in children aged 4–12 years similarly suggested that miscuffing may result in up to 5 mmHg differences of systolic BP measurements but no difference for diastolic BP with no influence of age and BMI (30). In our study, almost 40 percent of respondents indicated inappropriate upper arm cuff size selection, thus cumulatively increasing the risk of inaccuracy.

Incorrect positioning (i.e., not sitting straight, feet not resting on the floor, arm not relaxed, or upper arm cuff not placed at the same level as the heart (12) has been reported by 40 percent of respondents. Positioning may significantly affect BP measurement results, particularly an arm that is lower than the heart level can lead to overestimation of systolic BP by 3.7–23 mmHg (26). Another important issue is the selection of the method to measure BP. In our study, slightly more than half of the respondents reported that BP was measured using automated devices as opposed to auscultatory method. Currently, the ESH guidelines accept measurements with oscillometric devices as long as they have been validated in the pediatric population, but abnormal readings need to be confirmed by auscultation (8). However, we were unable to determine the devices used in individual cases and their validation status.

Finally, we also assessed whether the results of BP measurements are communicated to patients and found that feedback on the results is provided to approximately 40 percent of the patients. This means that more than half of the children and their families are left unaware of their BP status and, accordingly, potential life-style recommendations in high-normal or abnormal cases and as well showing that still BP measurements considered of low importance in children and adolescent health. To address discussed issues, Mitsnefes et al. proposed a systemic approach involving formal training, guideline education, dedicated teams, pre-visit planning, and electronic health records with high BP alerts to improve BP measurement practices and ensure timely follow-ups (31). Similarly, other studies have demonstrated that staff training, resource acquisition, and electronic health record alerts can enhance adherence to best practices in pediatric BP measurement (32, 33).

Our study is subject to several limitations. First, we evaluated BP measurement practices from the end-user perspective, thus their judgement on the appropriateness (compliance to the ESH guidelines) of BP measurement procedure may not be entirely accurate as they were not specifically trained or informed about the current clinical practice recommendations. In addition, as any survey it is at a higher risk of recall bias that depend on the respondent's beliefs, perception of BP importance, comorbidities and other factors. Similarly, the study is also subject to sampling bias due to potential of self-selection in the web survey part. The risk of this bias, however, is less pronounced in the adolescent survey where random selection procedure was used. Despite these potential limitations, large sample size and the review of the questionnaire by a pilot sample of respondents in the light of existing knowledge gaps regarding real-life practices of BP measurement constitute the strengths of our study.

In summary, our study exploring real-life practices of BP measurement in children and adolescents demonstrates insufficient rates of BP screening in the general pediatric population. Although the rates of BP screening increase with age, the compliance to available guidance remains insufficient, particularly in pre-school children. Importantly, only a very small fraction of children and adolescents appear to undergo BP measurements that do not include technical inaccuracies acting as potential sources of error. Collectively, this leads not only to potential under-recognition of abnormal BP in the pediatric population but also poses children and adolescents to inaccurate results and misclassification of BP status. Considering the relatively high level of evidence that constitutes the basis of current recommendations for BP measurement in children and adolescents, we suggest that future studies should focus on implementation research to improve adherence to best practices recommendations.

## Data Availability

The raw data supporting the conclusions of this article will be made available by the authors, without undue reservation.
