# Supplementary material for: Blood pressure measurement practices in children and adolescents within primary care setting
Source: Front Pediatr. 2025 Mar 21;13:1571419. doi: 10.3389/fped.2025.1571419 (PMC11968715; doi:10.3389/fped.2025.1571419)
Supplement: Supplementary file 1 [file Table1.docx]

Supplementary Materials

# Supplementary Data: Questionnaires

***Supplement 1.***

***Our questionnaire for parents consisted of the following questions:***

1. Which city or district of Lithuania do you live in? (Text answer)
2. How old is your child? Please enter a number. (Text answer)
3. What is the sex of your child? (Single choice)
4. Boy
5. Girl
6. Does your child have comorbidities such as diabetes mellitus; chronic kidney disease; congenital heart disease? (Single choice)
7. Yes
8. No
9. If you answered that your child has comorbidities, please specify which comorbidities your child has. (Multiple choice)
10. Diabetes mellitus
11. Chronic kidney disease
12. Congenital heart disease
13. Other (text answer)
14. Has your child been diagnosed with arterial hypertension (high blood pressure)? (Single choice)
15. Yes
16. No
17. Was your child born prematurely - premature or after was admitted to the neonatal intensive care unit? (Single choice)
18. Yes
19. No
20. Has your family doctor(s)/pediatrician(s) ever measured your child's arterial blood pressure at the clinic? (Single choice)
21. Yes
22. No

**If you answered Yes to question 8, please answer below:**

1. How often does your family doctor(s)/pediatrician(s) measure your child's arterial blood pressure? (Single choice)
2. Once a year (during pre-school/kindergarten check-ups)
3. Less than once a year
4. More than once a year

**Remember the last time when your child had his/her BP measured.**

1. Was the arterial blood pressure measured after the child was resting for 3-5 minutes? (Single choice)
2. Yes
3. No
4. Was it measured while sitting comfortably (i.e. with the child sitting upright, feet on the floor, relaxed with the arm and the upper arm cuff at the same level as the heart)? (Single choice)
5. Yes
6. No
7. Does your doctor(s) use a child-size cuff when taking measurements? Selects the right size cuff for the child according to the size of the upper arm. (Single choice)
8. Yes
9. No
10. Does your doctor(s) carry out multiple measurements during one visit? (Single choice)
11. Yes
12. No
13. Does your doctor(s) measure your child’s blood pressure "by ear" (i.e. using a stethoscope) or with an automated device? (Single choice)
14. Measures "by ear" (i.e. using a stethoscope)
15. Measures using automated device
16. Does your doctor(s) give feedback on your child's arterial blood pressure results? (Single choice)
17. Yes
18. No

***Supplement 2.***

***Our questionnaire for adolescents consisted of the following questions:***

1. Which city or district of Lithuania do you live in? (Text answer)
2. How old are you? Please enter a number. (Text answer)
3. Are you a girl or a boy? (Single choice)
4. Boy
5. Girl
6. Do you have comorbidities such as diabetes mellitus; chronic kidney disease; congenital heart disease? (Single choice)
7. Yes
8. No
9. If you answered that you have comorbidities, please specify which comorbidities you have. (Multiple choice)
10. Diabetes mellitus
11. Chronic kidney disease
12. Congenital heart disease
13. Other (text answer)
14. Have you been diagnosed with arterial hypertension (high blood pressure)? (Single choice)
15. Yes
16. No
17. Have you been born prematurely, or have you been admitted to the neonatal intensive care unit? (Single choice)
18. Yes
19. No
20. Has your family doctor(s)/pediatrician(s) ever measured your arterial blood pressure at the clinic? (Single choice)
21. Yes
22. No

**If you answered Yes to question 8, please answer below:**

1. How often does your family doctor(s)/pediatrician(s) measure your arterial blood pressure? (Single choice)
2. Once a year (during pre-school check-ups)
3. Less than once a year
4. More than once a year

**Remember the last time when you had your BP measured.**

1. Was the arterial blood pressure measured for you after resting for 3-5 minutes? (Single choice)
2. Yes
3. No
4. Was it measured while sitting comfortably (i.e. sitting upright, feet on the floor, relaxed with the arm and the upper arm cuff at the same level as the heart)? (Single choice)
5. Yes
6. No
7. Does your doctor(s) use a right size cuff when taking measurements? Selects the correct size cuff for you according to the size of the upper arm. (Single choice)
8. Yes
9. No
10. Does your doctor(s) carry out multiple measurements during one visit? (Single choice)
11. Yes
12. No
13. Does your doctor(s) measure your blood pressure "by ear" (i.e. using a stethoscope) or with an automated device? (Single choice)
14. Measures "by ear" (i.e. using a stethoscope)
15. Measures using automated device
16. Does your doctor(s) give feedback on your arterial blood pressure results? (Single choice)
17. Yes
18. No

# Supplementary Tables

***Supplement 3.***

**Table 3.** Parents’ and adolescents reported data: baseline characteristics

|  | **Parents’ reported data of their children** | **Adolescents’ reported data** |
| --- | --- | --- |
| **Characteristic** | **years** | **years** |
| **Mean age** | **5.1 ± 3.9** | **15.45 ± 1.14** |
|  | **n (%)** | **n (%)** |
| **Gender:** |  |  |
| Girls/ Boys | 711 (47.3)/ 793 (52.7) | 268 (59.8)/ 180 (40.2) |
| **Born premature or admitted to neonatal intensive care unit** | 194 (12.9) | 66 (14.7) |
| **Comorbidities:** | **70 (4.7)** | **34 (7.6)** |
| Congenital heart disease | 44 (62.9) | 27 (79.4) |
| Chronic kidney disease | 6 (8.6) | 1 (2.9) |
| Diabetes mellitus | 3 (4.3) | 6 (17.6) |
| Other (e.g. asthma, cystic fibrosis, hydronephrosis) | 17 (24.3) | 0 (0) |
| **Self-reported diagnosis of AH** | 15 (1) | 23 (5.1) |

***Supplement 4.***

**Table 4.** Parents’ and adolescents’ reported data: BP measurement practices in primary care setting

| **BP measurement peculiarities in primary care setting** | | |
| --- | --- | --- |
|  | **Parents’ reported data**  **of their children** | **Adolescents’ reported**  **data** |
| **Practice** | **n=498**  **n (%)** | **n=374**  **n (%)** |
| **BP measured** | 498 (33.1) | 374 (83.4) |
| **BP measurement frequency:** |  |  |
| Once a year (during preventive checks before school/ kindergarten) | 373 (74.9) | 298 (79.7) |
| Less than once a year | 69 (13.9) | 42 (11.2) |
| More often than once a year | 56 (11.2) | 34 (9.1) |
| **BP measured at rest** | 406 (81.5) | 223 (59.6) |
| **Appropriately sized upper arm cuff used** | 312 (62.7) | 219 (58.5) |
| **BP measured only one time during the visit** | 417 (83.7) | 293 (78.3) |
| **Incorrect positioning of the child during measurements** | 212 (42.6) | 143 (38.2) |
| **BP measured using automated device/ auscultatory method** | 279 (56)/ 219 (44) | 185 (49.5)/ 189 (50.5) |
| **Physician feedback on BP results to the parents and/or children** | 124 (24.9) | 223 (59.6) |
